# A knowledge graph based question answering method for medical domain

**DOI:** 10.7717/peerj-cs.667

**Published:** 2021-09-01

**Authors:** Xiaofeng Huang, Jixin Zhang, Zisang Xu, Lu Ou, Jianbin Tong

**Affiliations:** 1 School of Computer Science, Hubei University of Technology, Wuhan, Hubei, China; 2 Computer and Communication Engineer Institute, Changsha University of Science and Technology, Changsha, Hunan, China; 3 College of Computer Science and Electronic Engineering, Hunan University, Changsha, Hunan, China; 4 Hunan Province Key Laboratory of Brain Homeostasis, Third Xiangya Hospital, Central South University, Changsha, Hunan, China

**Keywords:** Knowledge graph, Medical domain, Question answering, Weighted path ranking

## Abstract

Question answering (QA) is a hot field of research in Natural Language Processing. A big challenge in this field is to answer questions from knowledge-dependable domain. Since traditional QA hardly satisfies some knowledge-dependable situations, such as disease diagnosis, drug recommendation, *etc*. In recent years, researches focus on knowledge-based question answering (KBQA). However, there still exist some problems in KBQA, traditional KBQA is limited by a range of historical cases and takes too much human labor. To address the problems, in this paper, we propose an approach of knowledge graph based question answering (KGQA) method for medical domain, which firstly constructs a medical knowledge graph by extracting named entities and relations between the entities from medical documents. Then, in order to understand a question, it extracts the key information in the question according to the named entities, and meanwhile, it recognizes the questions’ intentions by adopting information gain. The next an inference method based on weighted path ranking on the knowledge graph is proposed to score the related entities according to the key information and intention of a given question. Finally, it extracts the inferred candidate entities to construct answers. Our approach can understand questions, connect the questions to the knowledge graph and inference the answers on the knowledge graph. Theoretical analysis and real-life experimental results show the efficiency of our approach.

## Introduction

An intelligent question answering (QA) agent aims to first understand users’ intentions according to their questions in natural language, and then provide direct and precise answers. Compared with traditional information retrieval methods, QA approach offers a better way for information retrieval. Given a question, the QA will automatically take its rich consideration and directly return a correct answer to the user. Since traditional QA hardly satisfies some knowledge-dependable situations, such as disease diagnosis, drug recommendation, *etc*. In recent years, researches focus on knowledge-based question answering (KBQA), a QA process which has the ability of answering questions based on knowledge database, such as Lukovnikov et al. (2017), Qiu et al. (2018) and Yue et al. (2017).

Especially in medical domains, many researches proposed their medical KBQA. Some researches focus on case-based methods, such as Roberts et al. (2016), Guofei, Zhikang & Xing (2018), Yan & Li (2018), *etc*. They propose to match the questions to the similar sentences in historical cases which is supported by experts and recommend the cases as the answers corresponded to the questions. However, such case-based approaches have some problems: On one hand, the knowledge coverage area is limited by a range of historical cases. On the other hand, these methods need to compare each case in a serial manner to find the most similar answer, which takes a huge time. Some researches propose to extract topic, answer type from questions, and then discover several relevant articles based on their knowledge representations. After that, locating the answers in the articles both at document-level and paragraph-level, such as Goodwin & Harabagiu (2016, 2017), *etc*. However, the knowledge coverage area is also limited by a range of existed historical articles. Another researches such as Athenikos, Han & Brooks (2009) use description logic (*e.g*. OWL description logic) for answering medical question. The researches extract keywords and topics from questions to match the pre-defined answering logics. However, the pre-defined answering logics take too much human-labor, which limits the usage in a wide range of question answering.

To address these problems, we propose a knowledge graph based question answering (KGQA) method for medical domain. Unlike the above mentioned approaches, the idea of our approach is based on knowledge graph. To achieve the goal of our KGQA, there still exists some challenges:

- To build a knowledge graph for medical domains according to extensive documents, the challenge is how to establish relations among entities since there exist positive and negative relations and also exist strong and week relations.
- For understanding questions, the intentions of a question and connecting the questions to the knowledge graph are key problems we have to address. Questions in medical domains are domain restricted and deeply rely on knowledge ontology.
- For answering questions, the main challenge is how to precisely infer correct answers on a knowledge graph since the knowledge graph cannot directly give answers. Since the questions from users are not entire and not fully correct. For example, when users ask for disease diagnosis with their symptoms, they always miss some symptoms and surmise some symptoms but they are not really true. What’s more, the questions are diversified, to answer varied questions, a general inference method is needed.

Hence, we require a novel approach which can understand the questions, connect the questions to the knowledge graph and infer the answers based on the knowledge graph corresponding to the questions.

**Figure 1:**
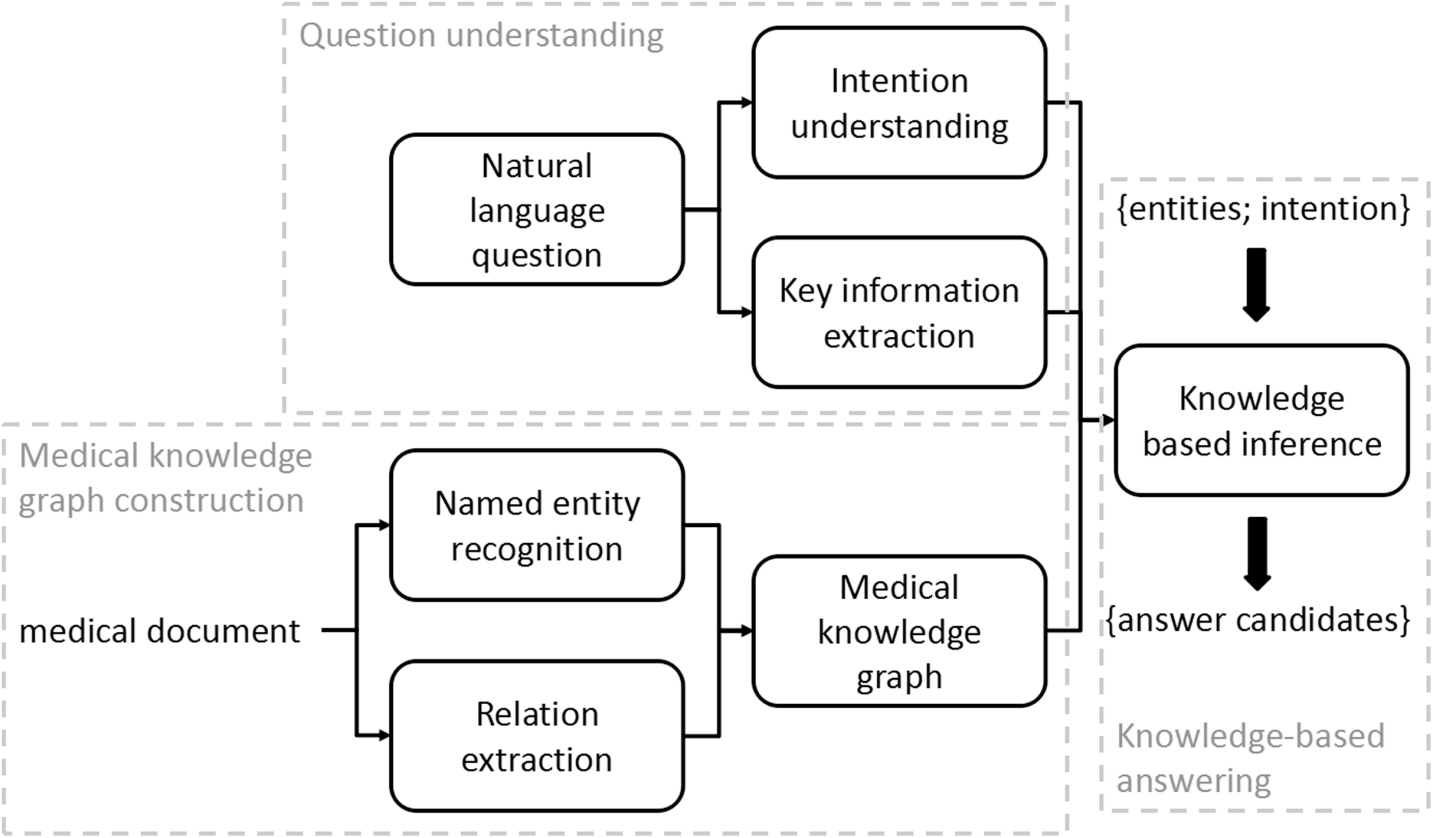
The work flow of our approach. Each corner matrix represents a key step/module in this approach, and the arrows represent the schedule of steps/modules.

The work flow of our KGQA is presented in Fig. 1. We first extract named entities and relations between the entities from documents. We then construct a knowledge graph, the vertices and the edges in the graph represent the entities and the relations respectively. Since the there exist positive and negative relations, based on text sentiment analysis, we assign positive and negative weights of edges to separately represent the two kinds of relations. For a given question, we extract the key information in the question based on the named entities, and meanwhile, we classify its intention by adopting information gain. Next, we connect the key information and the intention to the entities and the concept of the knowledge graph. For answering the question, we propose a weighted path ranking method based on the knowledge graph for scoring the related entities, and extract the inferred candidate on the knowledge graph to construct answers.

**Contributions** The main contributions of this paper are summarized as follows:

- In this paper, we propose a knowledge graph based question answering method for medical domain, which first constructs a medical knowledge graph, then understands users’ questions, and finally infers the answers on the knowledge graph corresponding to the questions.
- We construct a knowledge graph for representing the positive and negative relations among medical entities.
- To address the problem of knowledge graph based question understanding and answering, we propose a domain restricted question understanding approach and propose a weighted path ranking based inference on the knowledge graph.
- Theoretical analysis and real-life experimental results show that our approach performs well in terms of accuracy.

**Paper organizations** The remainder of this paper: In “Related Works” we introduce the related works. We propose our KGQA method for Medical Domain in “KGQA for Medical Domain”. Evaluations are shown in “Evaluation”. In “Discussion” we further discuss the advantages and defects of our approach. We conclude this paper and show the future work in “Conclusion”.

## Related Works

### Knowledge based question answering

Yue et al. (2017) proposed to bridge the gap between the given question and the answer entities by reconstructing the intermediate natural sequences on the basis of the entities and relations in knowledge bases. Qiu et al. (2018) detected topic entity to find out the main entity asked in a question, which is significant in question answering. They encoded question context, entity type and entity relation by LSTM. Yin et al. (2015) proposed to parse each paraphrased question into a set of tuple queries, and executed each tuple query against the open KB to obtain a list of candidate answers by using an alignment-based answer extraction. Li et al. (2017) considered the task of multi-modal question answering over structured data, in which a user supplies not just a natural language query but also an image by optimizing a non-convex objective function capturing multi-modal constraints. Wang et al. (2016) proposed to build a conditional knowledge base from user question-answer pairs for answering user questions with different conditions through dialogues. After that, they extracted the answers to questions conditioned on both question pattern clusters and condition clusters. Savenkov & Agichtein (2016) enriched question answering over a knowledge base by using external text data.

### Knowledge based question answering for medical domain

Duan, Yunbo Cao & Yu (2008) proposed a method for question analysis aimed at retrieving similar questions. The proposed approach identifies the focus and the topic of input questions. Similar questions are then retrieved by matching the extracted topic and focus. Roberts et al. (2016) discussed the relations between the three types of expected medical answers, namely the diagnosis, the tests, and the treatments as well as the medical findings pertaining to the medical case addressed by the clinical decision support topics. Guofei, Zhikang & Xing (2018) first classified and processed users’ questions into a sentence vector, then they adopted a matching algorithm to match the most similar question. After querying the constructed medical knowledge base, the corresponding answers to previous questions are responded to users. Yan & Li (2018) proposed a similarity calculation method based on vector space model and combining the weighted domain dictionary for health questions in QA. Goodwin & Harabagiu (2016, 2017) proposed a probabilistic representation of the medical knowledge by using a Markov network. Then they used the likelihood of the automatically discovered answers to produce several answer-informed rankings of the relevant scientific articles. And finally, they located the answers both at document- and paragraph-level.

### Knowledge graph

Knowledge graphs provide semantically structured information that is interpretable by computers. The knowledge graph techniques include knowledge representation, named-entity recognition and alignment, relation extraction and prediction, knowledge graph completion and validation, *etc*. Since in this paper, we construct a medical knowledge graph by structured raw data, the named-entities and relations are easy to extract, and this paper aims to build a question answering model based on knowledge graph, so here we mainly focus on the knowledge representation. Most knowledge graphs represent the knowledge by “entity-relation-entity” and are developed by RDF, OWL, *etc*. Some of them are automatically developed by natural language processing techniques such as named-entity recognition, relation extraction, *etc*. Recently, a large number of knowledge graphs have been created, including Suchanek, Kasneci & Weikum (2007), Auer et al. (2007), Carlson, Betteridge & Kisiel (2007), Bollacker et al. (2007). The Google Knowledge Graph (Singhal, 2012) is used to identify and disambiguate entities in text, to enrich search results with semantically structured summaries, and to provide links to related entities in exploratory search. The main tasks in knowledge graph construction include entity resolution (entity identification/recognition) and link prediction (Nickel et al., 2016).

### Path ranking

Lao et al. (2010) proposed a promising method for learning inference paths in large knowledge graph. Path-ranking uses a random-walk with restarts based inference mechanism to perform multiple bounded depth-first search processes to find relational paths. Xiong, Hoang & Wang, 2017 framed the path learning process as reinforcement learning (RL). They used translation based knowledge based embedding method to encode the continuous state of their RL agent. The agent takes incremental steps by sampling a relation to extend its path. Mihalcea & Tarau (2004) proposed TextRank, a graph-based ranking model for text processing, and showed how this model can be successfully used in natural language applications.

## KGQA for Medical Domain

### Overview of KGQA

In this paper, our KGQA include three key steps: knowledge graph construction, question understanding, knowledge graph based question answering. Our approach can formulate as *Answer = Inference*(*KG, Classifier*(*Question*), *Entity*(*Question*)), where *KG* is the knowledge graph, *Inference*() is the knowledge graph based inference method for question answering, *Classifier*() is the question classification method for recognizing the intention of a given question and *Entity*() is the entity extraction method for extracting the key information of a given question.

We first build a knowledge graph to make a structured representation of knowledge. Let *KG* = (*E, R, W, S, C*) be a knowledge graph, where *E* = {*e*_1_,*e*_2_,*…,e*_*n*_} is a set of entities, *R* = {*r*_1_,*r*_*2*_*,…,r*_*m*_} is a set of relations between two entities *e*_*i*_ and *e*_*j*_, *W* = {*w*_1_,*w*_*2*_*,…,w*_*m*_} is a set of weights corresponding to the relations, *S* = {*s*_1_,*s*_*2*_*,…,s*_*n*_} is a set of scores corresponding to the entities, and *C* = {*c*_1_,*c*_*2*_*,…,c*_*k*_} is a set of concept which the entities belong to.

Before answering a question, an intelligent agent should understand the question first. The intelligent agent should know the intention and the key information of the question. Here we build a classifier to learn the intention and extract the key information by searching the named entities in the knowledge graph. The intention and the key information will be used for inferring the answer on the knowledge graph *KG*. Let *E′ = Entity*(*q*_*i*_) be the method to extract the named entities of the question *q*_*i*_ where *E′* is the set of entities in *q*_*i*_. Let *c*_*k*_ *= Classifier*(*q*_*i*_) be the classifier to learn the intention of the question *q*_*i*_ where *c*_*k*_ is one of the concepts in the knowledge graph *KG*.

After we gain the concept *c*_*i*_ and key information *E′* of a question *q*_*i*_, the next we answer the question by a knowledge graph based inference method *a*_*i*_ *= Inference(KG, c*_*i*_, *E′)*. We propose a path ranking based inference method in which a score is associated with each entity in *KG* to represents the “correlation” of the entity corresponding to the question *q*_*i*_. After scoring each entity, we choose the entities related to *c*_*i*_ as candidates. The maximum scored entity will be used to generate an answer combining with the answer template.

### Medical knowledge graph construction

In this section, we need to construct a medical knowledge graph first since the graph can naturally represent the structured knowledge. We also assign the weight to each relation in the graph to describe the positive or the negative correlation.

Before we introduce the knowledge graph, we first describe raw data used to construct the knowledge graph. The raw data includes the medical instructions of drugs and diseases. The fields in the medical instructions of drugs include adaptation disease, adaptation symptom, taboo population, *etc*. and the fields in the medical instructions of diseases include complication symptom, body, hospital department, treatment, prevention, *etc*., as shown in Table 1. Each drug and disease have an instruction in which we can search the related entities, such as the symptoms related to a disease. We extract the entities of drugs, disease, symptoms, taboo population, *etc*. and connect them according to their common names. Besides, some field such as treatment and prevention will be treated as properties of an (disease) entity.

**Table 1:** The fields in medical instruction.

Drug	Disease
common name of drug	common name of disease
adaptation disease	complication symptom
adaptation symptom	body
taboo population	hospital department
sex	treatment
…	prevention
…	…

**Figure 2:**
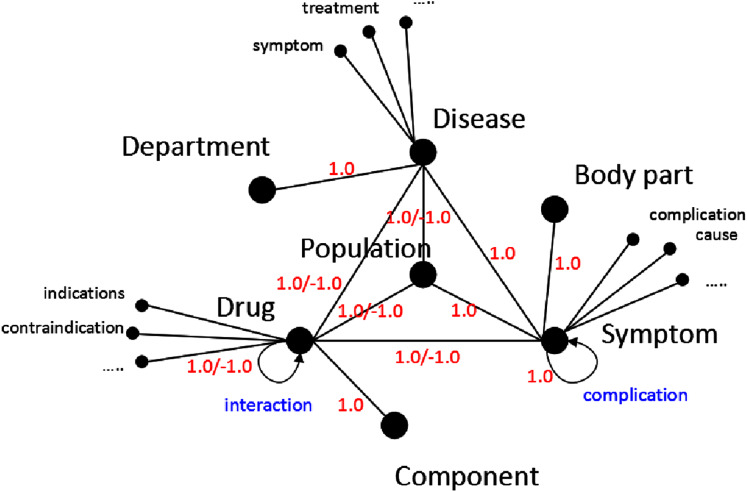
The medical knowledge graph in our approach. The bigger black circles represent the concepts of medial entities, the smaller black circles represent the properties of the entities and the edges represent the relations between two concepts. The scores marked in red of the edges represent the weights of the edges.

As shown in Fig. 2, the concepts of the medical entities include *disease*, *symptom*, *drug*, *sex*, *population*, *body part*, *etc*., so that the vertices in the knowledge graph also include disease entities (*e.g. cold*, *gastritis*, *etc*.), symptom entities (*e.g. cough*, *stomachache*, *etc*.), drug entities (*e.g. Aspirin*, *Cephalosporin*, *etc*.), sex entities (*e.g. man*, *woman*), population entities (*e.g. baby*, *pregnant woman*, *etc*.), body part entities (*e.g. head*, *chest*, *etc*.), *etc*. The properties of the disease entities include *symptom*, *treatment*, *etc*. The properties of the symptom entities include *prevention*, *cause*, *etc*. The properties of the drug entities include *indications*, *contraindication*, *etc*.

**Figure 3:**
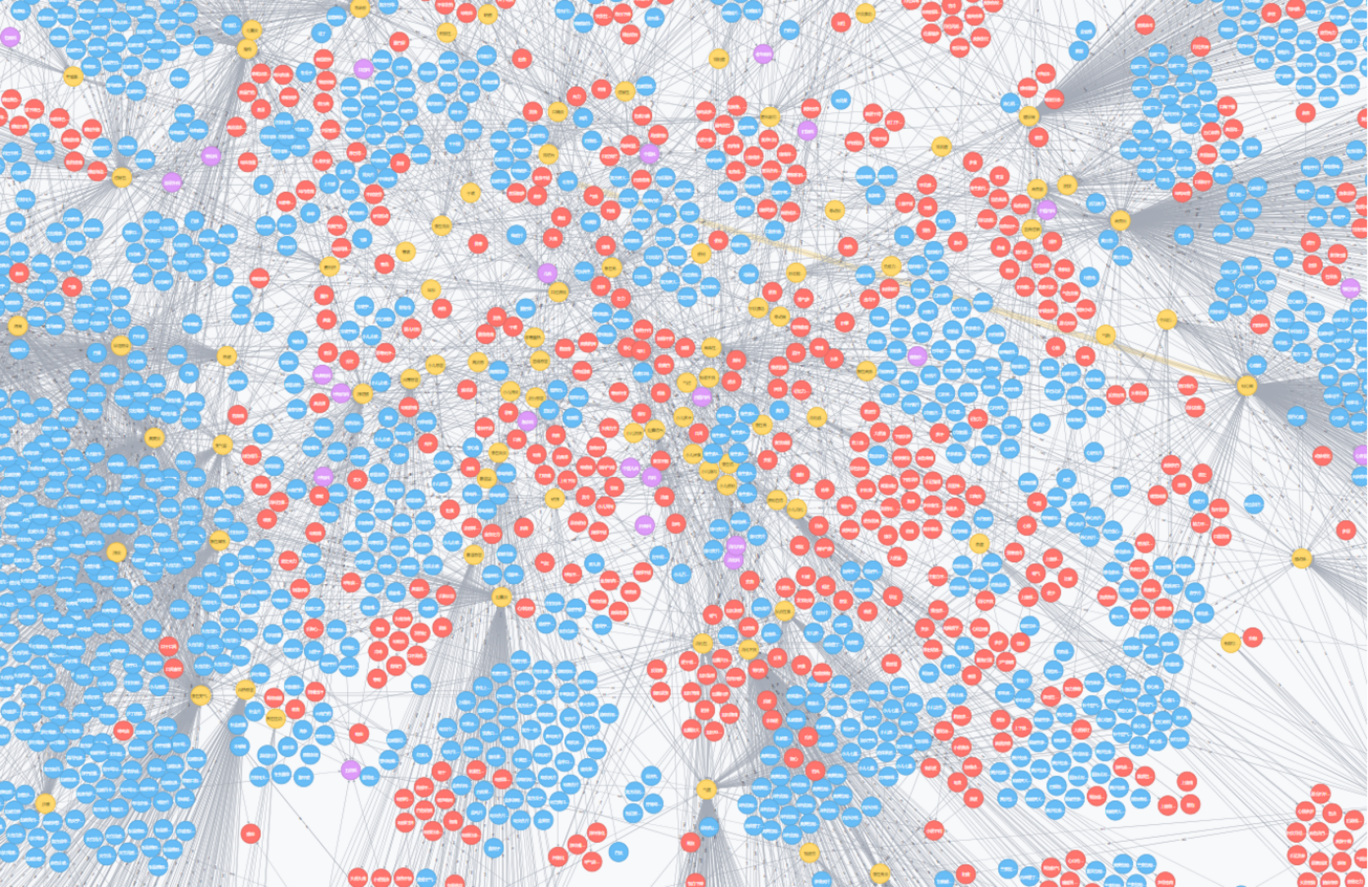
The medical knowledge graph. The red points represent symptoms, the yellow points represent disease, and the blue points represent drugs.

The edges in the graph represent the relations between two entities. For example, cold-cough, gastritis-stomachache represent cold and gastritis cause cough and stomachache, respectively. Since the relations between two entities include positive relations and negative relations, we assign positive weight (*1.0*) and negative weight −*1.0* to each edge, for example, the weight of the edge cold-cough is *1.0* because cold can cause cough, and the weight of the edge Tetracyclines-pregnant woman is −*1.0* because Tetracyclines is a taboo drug for pregnant woman. Since the relationships between a disease and its caused symptoms include strong correlations (main symptoms) and weak correlations (complications), to quantitatively distinguish the two kinds of correlations, we separately assign two initialized weight values *1.0* and *0.5* of the two correlations. As shown in Fig. 3.

A few researches propose to adopt statistical learning methods or neural networks for constructing a knowledge graph, such as Nickel et al. (2016). Although these methods are efficient, they still make some errors and miss many facts Xian et al. (2015). Since the knowledge graph is a base of our approach, it is very important to ensure the entities and relations in it are correct. Besides, the knowledge graph construction can be offline, which is not sensitive to the time consumption. We construct the knowledge graph only once, before we answer questions. Hence, we choose a semantic template matching based method which includes a set of hand-crafted rules to construct our medical knowledge graph. This method have high precision while compromising recall. The template designing costs several days of a designer.

The knowledge graph of entities and relations are automatically built according to medical instructions on *disease*, *symptoms*, *drugs* and *etc*. The data sets are collected from a medical company. Because most of medical instructions are expressional standard, so according to the key words which include a list of common names of *diseases*, *symptoms*, *populations*, *drugs* and *etc*., and semantic templates such as ([disease], [relation: “*cause/major symptoms … are*”], [symptom]), which formulate relations among components in sentences, we can extract entities and relations between entities from the instructions. To design the semantic templates, we first select a few sentences which contains entities and relations as seeds, and then we label the entities and relations in the sentences. The next, by summing up some patterns in the sentence, we save the patterns as rules to match a wide range of descriptions in the medical instructions.

For example, given an instruction “*Gastritis is inflammation of the lining of the stomach, …, the common is upper abdominal pain, nausea, vomiting …. Complications may include bleeding, stomach ulcers, …*” the disease vertices extracted from the instruction is {Gastritis}, the symptom vertices extracted from it are {Upper abdominal pain, Nausea, Vomiting, …, Bleeding, Stomach ulcers, …}, the relationships and weight values are {(Gastritis, Upper abdominal pain, 1.0), (Gastritis, Nausea, 1.0), (Gastritis, Vomiting, 1.0), …, (Gastritis, Bleeding, 1.0), (Gastritis, Stomach ulcers, 1.0), …}.

### Question understanding

In this work, to structurally represent a question, we need to extract the key information and recognize the intention of a given question.

To extract the named entities, we segment a list of words *WD* = {*wd*_1_,*wd*_2_,…*,wd*_*j*_*,…*} in *q*_*i*_ by using Jieba (2020) (delete the stop words, *e.g*. in English, “*is*”, “*has*”, “*and*”, “*of*”, *etc*.), a dictionary of named entities in the knowledge graph is used to match the named entities in the question *q*_*i*_. We search the words *WD* in the set of entities *E* in the knowledge graph *KG*, if *wd*_*j*_ can match any entity in *E*, then we add *wd*_*j*_ to *E′*.

The next, we adopt an information gain method to build the classifier which is used to learn the intention of the question *q*_*i*_. Since we can answer questions with a same intention in a same way, we convert this intention recognition problem to a question classification problem. The categories of questions which is also the concepts in our medical knowledge graph represent the intentions of questions, mainly include: “*disease diagnosis*”, “*symptom”*, “*treatment”*, “*diet*”, “*cause”*, “*drug recommendation*”, “*taboo population*”, “*indication*”, “*prevention*” *and extensions*. Since there are so many extensions, and in order to simplify the problem, we only classify questions into these nine types (exclude extensions). If a question does not belong to any of the nine types, we do not take it into consideration.

Let *c*_*k*_ be the *k*^*th*^ concept of the question. We represent a question as a set entities and a set of the other common words: *q*_*i*_ = {{*entity*}, {*word*}}. For entities, we use a general representation to represent entities in each different concept by replacing the entities in the question *q*_*i*_ to the concepts they belong to. A question contains an entity $e_j^{\prime}$ which belong to a concept *c*_*k*_, we replace the entity $e_j^{\prime}$ to the concept *c*_*k*_, so the *WD* is replaced to *q*_*i*_ = {{*concept*}, {*word*}}. For example, we use *@disease* to represents all entities in disease concept (*e.g*. cold, heart disease, *etc*.), and we use *@drug* to represents all entities in drug concept (*e.g*. Aspirin, Cephalosporin, *etc*.). Given a question *q*_*i*_ in English “*How to prevent a cold?*”, we represent it as *q*_*i*_ = {*how, prevent, @disease*}.

We address the problem to a short text classification problem and address the problem by using a Information Gain based method *IG*(*x*_*j*_, *c*_*k*_) according to Eq. (1). We calculate the information gain for each element {*concept*}, {*word*} in all of the questions and for each concept *c*_*k*_ in knowledge graph, where *x*_*j*_ is the element in questions. The classification model is used for identifying what category a question belongs to. For example, some questions ask for disease diagnosis which belongs to disease diagnosis category, some questions ask for the treatment of a disease which belongs to treatment category, *etc*. We first extract the medical entities and other words (after deleting the stop words) as the features and adopt one-hot vector to represent the features, then we send the features into the classification model and train the classification model by using Information Gain. In the train process, based on the training data, we use the Eq. (1) to calculate the information gain of each feature corresponding to each category. The information gain represent the importance of each feature corresponding to each category. After training, we can sum the total value according to Eq. (2), which means the probability of a question belonging to each category, and then find the category which has maximum value as the category that the question belongs to

(1) $$IG({x_j},{c_k}) = p({x_j}|{c_k}) \cdot In \left(\displaystyle{{p({x_j}|{c_k})} \over {p({x_j}) \cdot p({c_k})}}\right)$$

The *p*(*x*_*j*_*|c*_*k*_) represents the probability of *x*_*j*_ belongs to *c*_*k*_, the *p*(*x*_*j*_) represents the probability of *x*_*j*_ in all of the questions and *p*(*c*_*k*_) represents the proportion of the questions of *c*_*k*_. If *x*_*j*_ is highly related to *c*_*k*_, then $In\left(\frac{p({x_j}|{c_k})}{p({x_j}) \cdot p({c_k})}\right)$ will be much more than zero. Otherwise, If *x*_*j*_ is independent of *c*_*k*_, then $In\left(\frac{p({x_j}|{c_k})} {p({x_j}) \cdot p({c_k})}\right)$ will be very close to zero.

After gaining *IG*(*x*_*j*_, *c*_*k*_), we sum the total value of *x*_*j*_ in a given question *q*_*i*_ for each concept, according to Eq. (2). If a concept *c*_*max*_ has maximum value, then *c*_*max*_ will be the concept of the question which represents the intention of the question.

(2) $$\matrix{ {value({x_j},{c_k}) = \sum\limits_{{x_j} \in {q_i}} {x_j} \cdot IG({x_j},{c_k})} \hfill \cr }$$

The pseudo-code of our intention recognition method is presented in Algorithm 1.

**Algorithm 1:** Information gain based question intention recognition.

**Input:** A representation of a question: *q*_*i*_ = {{*entity*}, {*word*}}, where {*entity*} is a set of entities in *q*_*i*_, and {word} is a set of the other common words in *q*_*i*_.
**Output:** The concept of the question *q*_*i*_: *c*_*max*_.
1: Replace the {*entity*} in *q*_*i*_ to {*concept*} which the {*entity*} belongs to.
2: Calculate the information gain for each element *x*_*j*_ in *q*_*i*_ for each concept *c*_*k*_ according to *IG*(*x*_*j*_*,c*_*k*_).
3: **function** *RecogConcept*(*q*_*i*_)
4: Scan *x*_*j*_ in a given question *q*_*i*_;
5: Sum the total value of *x*_*j*_ in the given question *q*_*i*_ for each concept *c*_*k*_;
6: Find a concept *c*_*max*_ which has a maximum value;
7: **return** *c*_*max*_.
8: **end funtion**

### Knowledge graph based question answering

Once we have constructed our knowledge graph, then we propose our knowledge graph based inference method in this Section. In the knowledge graph, the procedure of answering a question can be transformed into a traversal that starts from the multi-entities in a question and searches for the appropriate path to reach the entities in a answer.

We first formulate the problem. Let *q*_*i*_ = {*e*_1_,*e*_2_,…*,e*_*n*_*,c*_*k*_} be the set of entities in the question *q*_*i*_, where *e*_*j*_ is the entities and *c*_*k*_ is the concept of the question. Let *KG* be the knowledge graph, the purpose of the inference method *I*(*q*_*i*_, *KG*) is to search the most appropriate entity as the candidate for constructing answer corresponding to the question *q*_*i*_.

In recent years, the Path-Ranking Algorithm (PRA) Lao, Mitchell & Cohen (2011) emerges as a promising method for inference in knowledge graphs. PRA uses a random-walk mechanism to search and score relational entities. Here we design a weighted scoring method for our path ranking based inference *I*(*q*_*i*_, *KG*) on knowledge graph.

Let *WScore*(*e*_*i*_) be the weighted score of the specific entity *e*_*i*_, according to Eq. (3), where *In*(*e*_*i*_) is the in-degree of *e*_*i*_, *Out*(*e*_*j*_) is the out-degree of *e*_*j*_, *w*_*j,i*_ is the weight of the edge between *e*_*i*_ and *e*_*j*_, and *α* = 0.85 is an experience parameter.

(3) $$\matrix{ {WScore({e_i}) = (1 - \alpha ) \cdot \alpha \cdot \sum\limits_{{e_j} \in In({e_i})} \displaystyle{{{w_{j,i}}} \over {\sum\nolimits_{{e_k} \in Out({e_j})} {w_{j,k}}}} \cdot WScore({e_j})} \hfill \cr }$$

We initially assign *1.0* as a score for each entity of the question *q*_*i*_ in the knowledge graph *KG*. Then we iterates the above scoring method until convergence variance below a given threshold. By running the algorithm, each entity in the knowledge graph has a score which represents the “importance” of the entity. After scoring the entities, we have a ranked list of entities with their scores. We only choose the entities related to the concept of the question *q*_*i*_ as candidates. The maximum scored entity will be used to generate an answer combining with the answer template.

For example, if a question is to ask for disease diagnosis, the maximum scored entity is “*cold*”, a template will be selected for answering disease diagnosis, like “*According to your descriptions, your most possible illness is a [disease]*”, then the answer to the question is “*According to your descriptions, your most possible illness is a cold*”.

The pseudo-code of our inference method is presented in Algorithm 2.

**Algorithm 2:** Path ranking based inference method on medical knowledge graph.

**Input:** A set of entities of a given question *q*_*i*_: *q*_*i*_ = {*e*_1_,*e*_2_,…,*e*_*n*_,*c*_*k*_}, where *e*_*j*_ is the entities and *c*_*k*_ is the concept of the question; A constructed knowledge graph *KG*.
**Output:** The maximum scored entity *e*_*max*_.
1: Assign *1.0* as a initial score for each entity of the question *q*_*i*_ in the knowledge graph *KG*, the initial scores of the other entities in *KG* are *0.0*.
2: **function** *I*(*q*_*i*_, *KG*)
3: **while:** Total convergence variance of the entities in *KG* is larger than a threshold *t* **do**
4: Randomly choose a entity *e*_*i*_;
5: Calculate the score of *e*_*i*_ according to Eq. (3);
6: Update the convergence variance;
7: **end while**
8: Gain a ranked list of entities with their scores;
9: Only select the entities which is corresponded to the concept of question *q*_*i*_;
10: Leave the maximum scored entities *e*_*max*_;
11: **return** *e*_*max*_.
12: **end function**

Based on the above weighted path-ranking method, we can infer answers on knowledge graph. Besides, the questions from users belong to different categories. For some categories, such as *symptom*, the users want to know the symptoms of a given disease. The disease entity and its symptoms have already been connected in the knowledge graph. So for these questions, we can obtain the answers by searching in the knowledge graph directly. This way is to find the answer by gaining the properties of the entities. For example, given a question “*What are the symptoms of a cold*”, we can directly get the answer by search the property “*symptom*” of disease entity “*cold*” on the knowledge graph, where the “*symptom*” is the concept of the question and the “*cold*” is a entity in the question. Choosing the weighted path-ranking method or the linked method can be pre-defined based on the concept of the question.

## Evaluation

In this section, we perform several experiments to evaluate our KGQA approach. We first introduce the experimental setup, data set and validation, then we show the topological property evaluations of our medical knowledge graph, the performance evaluation of our question understanding method and the performance evaluation of our knowledge graph based question answering, finally we introduce a cased study on how our KBQA works for medical domain.

### Experimental setup

We implement our experiments on one computer. The version of its CPU is Intel i5-3470@3.20 GHz, the RAM is 16.0 GB and the operation system is Linux Ubuntu 16.04. Our medical KBQA is developed by Java programming language. We collect the raw data from a medical company named YiFeng Pharmacy (2020, http://www.yfdyf.cn/) and store the raw data by MySQL 5.7.

We first represent the knowledge graph data by entity-relation-entity as that in RDF, then our weighted path-ranking method will load a part of knowledge graph data (the connected entities and relations corresponding to the questions) in memory for answering the questions of disease diagnosis, and finally import the knowledge graph data into the graph database Neo4J for visualization as shown in Fig. 3. The raw data set we used to build disease-symptom graph includes *182,638* standard medical instructions on *drugs*, *disease* and *etc*. These instructions are collected from the medical company YiFeng Pharmacy (2020, http://www.yfdyf.cn/). The original data we collected is in Chinese.

We also collect *149,998* records of medical question-answer pairs of users from YiFeng, which include nine categories: “*disease diagnosis”, “symptom”, “treatment”, “diet”, “cause”, “drug recommendation”, “taboo population”, “indication” and “prevention”*, as shown in Table 2.

**Table 2:** The number of question-answer pairs in different categories.

Category	Number
disease diagnosis	1,612
symptom	22,849
treatment	36,800
diet	14,505
cause	9,842
drug recommendation	2,674
taboo population	25,614
indication	19,681
prevention	16,421
total	149,998

For question answering, the questions in categories of “*disease diagnosis*” will be sent to our weighted path ranking method to diagnose diseases and recommend drugs. The answers for the questions in other categories will be searched according to the relations between entities and their properties. To validate the results, the categories and answers (disease entities or drug entities) of the questions are prepared by YiFeng.

### Topological property evaluations of our medical knowledge graph

The medical knowledge graph contains 8,868 disease entities, 5,895 symptom entities 19,448 drug entities, 520 population entities, 55 body part entities, *etc*. and 601,475 relations among the entities. Since the average number of neighbors and the graph density can represent a knowledge graph is dense or sparse, we use these metric to show the global topology properties of our knowledge graph, as shown in Table 3. The average number of neighbors and the graph density show that the entities in the knowledge graph are highly connected.

**Table 3:** The global topology properties of our knowledge graph.

Item	Value
Total nodes	34,788
Total edges	601,475
Average number of neighbors	34.58
Graph density	9.94 × 10^−4^

Our medical knowledge graph is partially presented in Fig. 3. The red points represent symptoms, the yellow points represent disease, and the blue points represent drugs. As we observed in this figure, we can easily find that the entities are significantly clustered, which means the neighbors of an entity may be used for inferring the related entities. The disease entities cluster the symptom entities and drug entities that conforms to medical facts.

### Performance evaluation of our question understanding method

Here we evaluate the performance of our question understanding method. The metric we used include: training accuracy, decision accuracy, training time cost and decision time cost. The proportion of $\textstyle{{{N_{training}}} \over {{N_{decision}}}}$ is *1*.

The performance of our method is presented in Table 4. The results show our method achieves *94.94%* of training accuracy and *93.89%* of decision accuracy, which demonstrate our method performs well in terms of accuracy. The results also show our method costs *201.08 s* of training time for all of the questions in training set and costs less than *0.001 s* decision time for each question in decision set, which means our method performs fast enough.

**Table 4:** The performance evaluation of our medical question understanding method.

Benchmark	Result
Training accuracy (%)	94.94%
Decision accuracy (%)	93.89%
Training time cost (s)	201.08 s
Decision time cost (s)	<0.001 s

**Figure 4:**
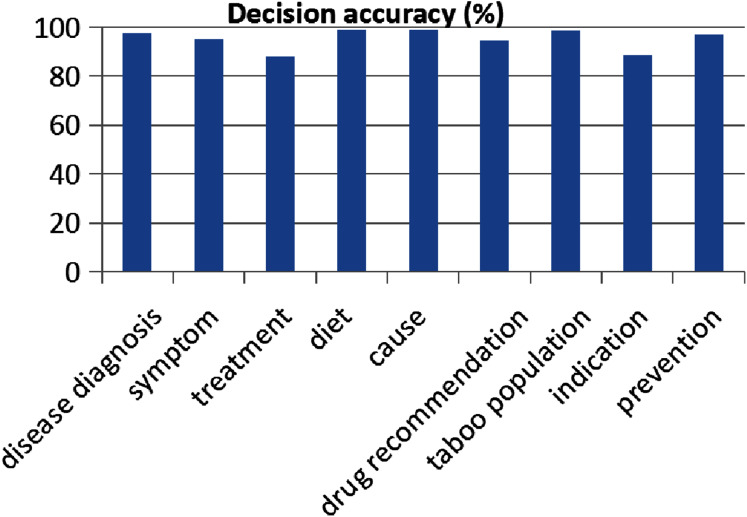
The accuracy evaluation of our question intention recognition method for different question types.

In addition, we show the decision accuracy in different categories: “*disease diagnosis*”, “*symptom*”, “*treatment*”, “*diet*”, “*cause*”, “*drug recommendation*”, “*taboo population*”, “*indication”* and “*prevention*”, as shown in Fig. 4. The results show our method performs well in all of question categories.

### Performance evaluation of our knowledge graph based question answering

For given a question, our approach answer the question in two ways: one is to directly search properties of entities in the question, which covers the question categories of “*symptom*”, “*treatment*”, “*diet*”, “*cause*”, “*taboo population*”, “*indication*”, “*prevention”* and “*drug*”, the other is to infer entities through our weighted path-ranking method with the entities in the question, which covers the question categories of “*disease diagnosis*”.

For answering the question from “*disease diagnosis*”, we initially assign the score of the entities in our knowledge graph to *0.0*, and assign the score of the entities in a given question to *1.0*. After that, we iterate our weighted path-ranking method to score the related disease and drug entities. A disease entity which has a maximum score (among disease entities) will be the result for disease diagnosis and a drug entity which has a maximum score (among drug entities) will be the result for drug recommendation. Finally, we embed the inferred results into prepared template to generate answers.

To show our method is efficient, we implement several state-of-the-art disease diagnosis methods for comparisons in the experiments. Since Bayesian is a widely used reasoning solution of joint probability distributions over a set of entities, Vembandasamy (2015) presented a work to diagnose diseases by using Bayesian algorithm. It is based on a simple assumption that each entity are independent with each other. Salem (2007) proposed to record and index past cases, then search them to identify the ones that is similar to new cases, the diagnosis of most similar past case will be useful for new cases.

In our experiments, the features are extracted for question answering includes the entities in the knowledge graph, such as symptom entities. We represent the feature by using one-hot vector. All of the methods (includes our approach and the other methods) use all of the same entities as the features for comparison. In our approach, we use the features ranking in the knowledge graph to diagnose disease. In other methods, one is to input the features into a Bayesian model and the other one is to search similarities based on these features.

Table 5 shows the performance comparisons of our approach and the other state-of-the-art methods. From the results, we can easily find out that our approach is comparable in terms of accuracy: the accuracy of our approach is *86.4%* while the accuracy of Vembandasamy et al.’ approach is *78.1%* and Abdel-Badeeh et al.′ approach is *75.2%*. The disease coverage means the coverage rate of disease categories that are correctly diagnosed once. The results show that our approach covers the most disease categories by comparing with the other state-of-the-art methods.

**Table 5:** The performance evaluation of our method and the other state-of-art methods for disease diagnosis.

Method	Decision accuracy (%)	Disease coverage (%)
Our approach (KGQA)	86.4	95.7
Vembandasamy et al. (Bayesian)	78.1	92.4
Abdel-Badeeh et al. (Case based)	75.2	90.3

For answering the questions from other categories, we test our approach with the cases in which we correctly extract entities and concept of questions. Our approach achieves *100%* accuracy when assuming the information in our knowledge graph is correct. For example, a given question is “*What’s the symptoms of a cold?*”, we only need to search the symptoms according to a disease entity “*cold”*.

### A case study

Here we present a simple case to show how our method works. As shown in Table 6, for a given question “*My child has a cough, a pectoralgia, a shiver and a fever recently. What disease does my child have?*”, the entities we extract from the question are {*child, cough, pectoralgia, shiver, fever, disease*}. After that, we convert the question to a structured text {*@population, has, @symptom, @symptom, @symptom, @symptom, recently, what, disease, @population, have*}, we use *@population* to represents all entities in population concept and use *@symptom* to represents all entities in symptom concept. Then we classify the question into the category *disease diagnosis*. The next, by using the knowledge graph based inference method, we infer *Pneumonia* as the result of the disease diagnosis. Finally, according to the template, we generate the answer “*Hello, we think it perhaps be a Pneumonia. The main symptoms of Pneumonia include productive cough, pectoralgia, fever, shiver and trouble breathing*.”

**Table 6:** The case study of our approach.

Item	Content
Question	“My child has a cough, a pectoralgia, a shiver and a fever recently. What disease does my child have?”
Named entity	{child, cough, pectoralgia, shiver, fever, disease}
Structured text	{@population, has, @symptom, @symptom, @symptom, @symptom, recently, what, disease, @population, have}
Category	disease diagnosis
Inference result	Pneumonia
Template	“Hello, we think it perhaps be a [@Disease], The main symptoms of [@Disease] include [@Disease]-[@Symptom].”
Answer	“Hello, we think it perhaps be a Pneumonia. The main symptoms of Pneumonia include productive cough, pectoralgia, fever, shiver and trouble breathing.”

## Discussion

In this section, we further discuss the advantages and defects of our approach.

By comparing with the case based KBQA, one of the most important advantages is that our approach does not limit to the range of historical cases, it can be applicable for all possible decision routes on knowledge graph and can infer some decisions where do not exist in historical cases. In addition, our approach can not only be used for medical domain, but can also be used for other knowledge-dependable domains, such as chemistry, biology, education, *etc*.

Although theoretical analysis and real-life experimental results show that our approach is efficient, it still remain a limitation: since our approach can only provide one answer once a process, our approach cannot handle questions with multi-intentions. Different intentions need different inference process.

We collected the data from a medical company. Although the medical data has been validated by some pharmacists and doctors, there may exist some potential data quality issues such as manual mistakes, ambiguity of medical knowledge, *etc*. Which may impact on the effectiveness of our approach.

In addition, knowledge graph validation is very important for the effectiveness of applications (Färber et al., 2018; Debattista et al., 2018). The knowledge graph can be validated based on three aspects including: accuracy, consistency and conciseness (Zaveri et al., 2016). Since the knowledge graph validation is an advanced field and needs further researches, so we consider it as the future work to improve the quality of our medical knowledge graph.

## Conclusion

In this paper, we propose a knowledge graph based question answering approach for medical domain. In our approach, we first construct a knowledge graph which contains medical entities, properties of the entities, relations among the entities and the weights of the relations. Then we recognize the concept and entities of a specific question. The concept and entities in the question will be sent to an inference method. Finally, we propose a weighted path ranking method for inferring on the knowledge graph to find an appropriate answer. The real-life experimental results demonstrate that our approach is efficient.

As a future work, we will consider to answer the question with multi-intensions which propose to divide different intensions in the question and provide appropriate answers for different intensions.

## Supplemental Information

10.7717/peerj-cs.667/supp-1 Supplemental Information 1 The code for constructing our medical knowledge graph, including collect the raw data from the Internet, handle the raw data and build a graph of entities and relations.

10.7717/peerj-cs.667/supp-2 Supplemental Information 2 The disease entities, symptom entities and their relations in the knowledge graph.

## Additional Information and Declarations

### Data Availability

The following information was supplied regarding data availability: The code and the data to build a medical knowledge graph are available as Supplemental Files.
